# Fluorescence-Guided Raman Spectroscopy with an Integrated Adapter for Faster and Cost-Effective Microplastic Detection

**DOI:** 10.1021/acs.analchem.5c04637

**Published:** 2025-11-20

**Authors:** Anna Kukkola, Liam Kelleher, Iseult Lynch, Stefan Krause

**Affiliations:** † School of Geography, Earth and Environmental Sciences, 1724 University of Birmingham, Edgbaston, Birmingham B15 2TT, United Kingdom; ‡ Ecologie des Hydrosystèmes Naturels et Anthropisés (LEHNA), Université Claude Bernard Lyon 1, Lyon, Villeurbanne 69622, France

## Abstract

Microplastic (MP) research is a rapidly evolving field, with their presence extensively evidenced across all environmental and biological matrices. However, the lack of standardized detection techniques in MP studies presents challenges in obtaining consistent and reliable results. This study introduces an integrated analytical approach within a single instrument, enhancing efficiency and accuracy in MP analysis. A cost-effective adapter was developed to seamlessly combine fluorescence staining with Nile Red and Raman spectroscopy for MP identification and polymer type determination. The system’s principle and performance were evaluated using ten different stained polymer types, demonstrating compatibility with both measurement modalities without interference. The validity was assessed by using environmental samples, including river and drinking water samples, which were analyzed using the system. An average reduction of 84 ± 8% (33 min) in analysis time was achieved, with 87 ± 6% of MPs correctly identified (19 out of 22 spectra measured) in comparison to measurements without fluorescence guided prescreening, where only 9 ± 3% of MPs were identified (10 out of 108 spectra measured). This approach improved analytical accuracy due to prescreening selectivity with fluorescence, leading to measurement times being drastically reduced. These findings suggest that the proposed system offers a significant cost-effective advancement for laboratories using Raman spectroscopy, enabling greater selectivity and higher sample throughput.

## Graphical abstract

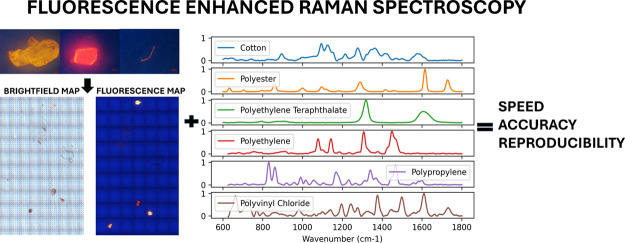

## 1 Introduction

At present, no universally established standard analytical technique exists for microplastics (<5 mm) in any environmental matrix,^1^ where processes can be time-consuming and difficult. Common methods for the isolation of microplastics (MPs) from environmental matrices before analytical quantification include density separation with salt solutions (e.g., NaCl or ZnCl_2_), and chemical/enzymatic digestion.^2,3^ While brightfield microscopy is widely used for counting MPs,^4^ it is unreliable and subjective for particles <200 μm.^5–7^ To reduce visual bias, fluorescence staining, with fluorophores such as Nile Red (NR), has gained attention.^8–12^ NR binds to plastics due to its hydrophobicity, making the plastics brightly fluorescent under specific wavelengths, while organic matter stains poorly, improving detection efficiency.^10,13^ However, false positives may still occur without proper quality assurance, including fluorescence intensity thresholds,^14,15^ and further chemical validation is recommended for at least 30% of suspected plastic particles to ensure accurate representation of MP numbers.^16^

Chemical validation of a subset of suspected MPs often involves manual sample transfer to a new vial or filter to use techniques like Raman or FTIR spectroscopy, introducing size-dependent limitations (due to filters used or limitations of manual picking of particles) and the potential for false negatives due to subsampling. Although there is much work on subsampling filters for MP analysis, there is no agreement on the best approaches and many agree that while the approach saves time it is prone to increasing false negative results, given that a partial measurement is made that can underestimate MP numbers. Larger particles are easier to manipulate, whereas smaller particles risk being contaminated with nonfluorescent debris, requiring count corrections. Scanning entire filters can mitigate this but is time-intensive and costly. Subsampling filters has been proposed but may introduce significant errors due to MP heterogeneity.^17^

To overcome these issues and to ensure that only fluorescent particles are included in the chemical verification step, new procedures are urgently required to aid the detection of the particles of interest more accurately. Several studies have proven the effectiveness of Nile red for fluorescence identification of MPs, and validated their findings with RS.^12,18^ However, only one study has explored the combination of fluorescence mode and Raman spectroscopy,^19^ although here an external hand-held forensic light was used in combination with an orange filter to preidentify areas of interest for subsequent inspection with Raman. This approach may lead to bias, as only the very bright particles may be seen via the naked eye and some smaller particles may not be identified in the prescreening phase. To overcome this issue, the Raman system itself needs to be modified so that it can facilitate the identification of the fluorescent particles in fluorescent mode.

Here we introduce an advanced, cost-effective method to enhance existing Raman systems for fluorescence-guided MP identification. By integrating NR staining in fluorescence mode and Raman spectroscopy analysis, the modified system improves the identification, removes the need to transfer samples, and saves time spent on imaging each filter/sample. This innovation addresses challenges related to false negatives from subsampling, size limitations due to manual handling and instrument time/costs, thus streamlining the detection process for more accurate overall MP analysis.

## 2 Materials and Methods

### 2.1 Reference Microplastics

Twelve reference MPs, previously identified by thermogravimetric analysis-Fourier transform infrared spectroscopy-gas chromatography–mass spectrometry,^20^ were prepared. These included high-density polyethylene (HDPE, opaque), polyamide-6 (PA-6, transparent), polyethylene (PE, transparent), expanded polystyrene (EPS, white), polyethylene terephthalate (PET, transparent), polyester (PES, transparent/blue), polylactic acid (PLA, opaque), three polypropylene (PP) variants (stiff and soft: gray, white and blue), polystyrene (PS, transparent), and polyvinyl chloride (PVC, white). The macroplastics were ground into smaller fragments using a spice mill, and dry sieved to a size range of into 64–400 μm. Each MP type was suspended in 20 mL of deionized water in separate glass beakers and stained with 5 μg/mL NR stock solution in acetone for 1 h at ∼21 °C following the guidance in.^13^ The stained particles were then vacuum filtered onto a Whatman GF/D glass fiber filter.

### 2.2 Environmental Samples

Eleven water samples were randomly selected from prior MP studies, fragments and fibers >64 μm. Six river water samples from the River Blythe^21^ were prepared on Whatman Anodisc filters (pore size 0.2 μm). Five drinking water samples from public sources in Mali^22^ were prepared on Whatman GF/D filters (pore size 2.7 μm). After the initial studies, the NR-stained MPs were stored in sealed containers within dark metal cabinets to prevent degradation of the NR dye from light exposure before analysis.

### 2.3 Fluorescence and Brightfield Light Adapters for Raman

The adapter was produced by modeling in 3D design software Autodesk Fusion 360 (Figure 1), design schematics are noted in Figures S1–S3. The adapter supports both fluorescence and brightfield modes and was developed to fit standard microscopy lamphouse ports (this includes models from Renishaw, Horiba, Thermo Scientific, PerkinElmer and Bruker). The design includes both straight-through and 90-degree geometries, with a silica wafer acting as the mirror due to its slim profile. While not an issue, silica has a poor reflectance value in the visible to near-infrared region, ranging from 30 to 40%.^23^ As an alternative a plano aluminum mirror could be used, however, they are more costly, and thicker, requiring design adjustment.

**Figure 1:**
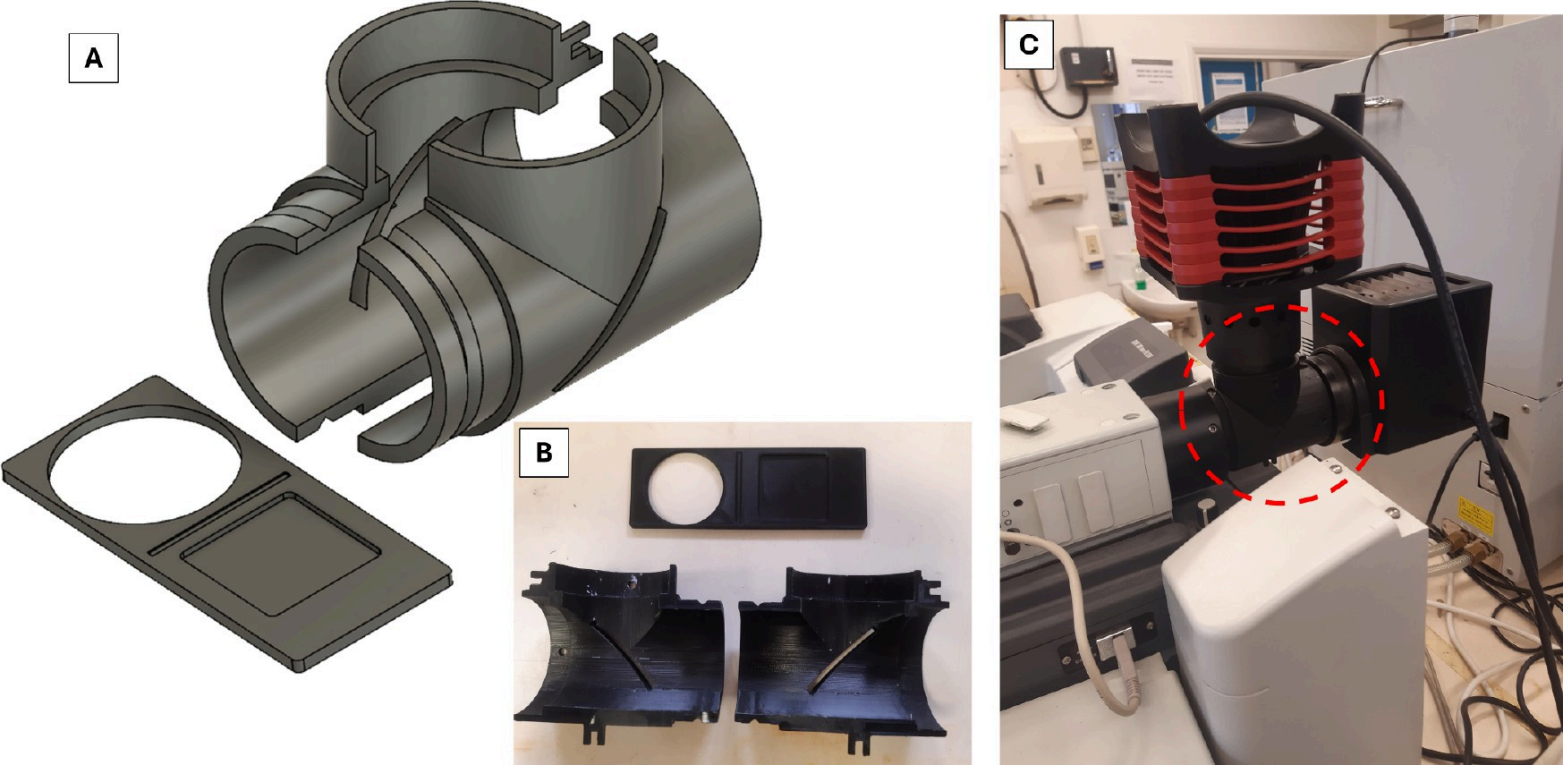
(A) Rendered image of the fluorescence and bright light source adapter designed for standard (Raman) microscope ports and fittings. (B) Image of the 3D-printed holder and mirror slider, produced in two pieces for ease of printing. (C) Photograph of the assembled holder (in red dashed circle) with a Thorlabs Solis LED (set to 50%) and an Olympus TH4–200 halogen light source (set to 40% for Anodisc and 50% for GFD), showing the complete setup mounted on a Thermo Scientific DXR2 Raman Microscope.

### 2.4 Fluorescence and Raman Spectroscopy

Two Raman microscope systems, a Renishaw InVia 2000 and a Thermo Scientific DXR2, were tested with the adapter, Figure 2 depicts the optical path used in such microscope-based systems that image MPs. Fluorescence measurements were performed using a Thorlabs Solis 470 nm LED light with a driver and a Thorlabs 505 nm long-pass dichroic filter. The full parts list and costs to install the modification are provided in Table S2. Bright-field measurements were conducted using a halogen light source (Olympus TH4–200). Both light sources were transferred between the systems, but objectives and other microscope components were not shared.

**Figure 2:**
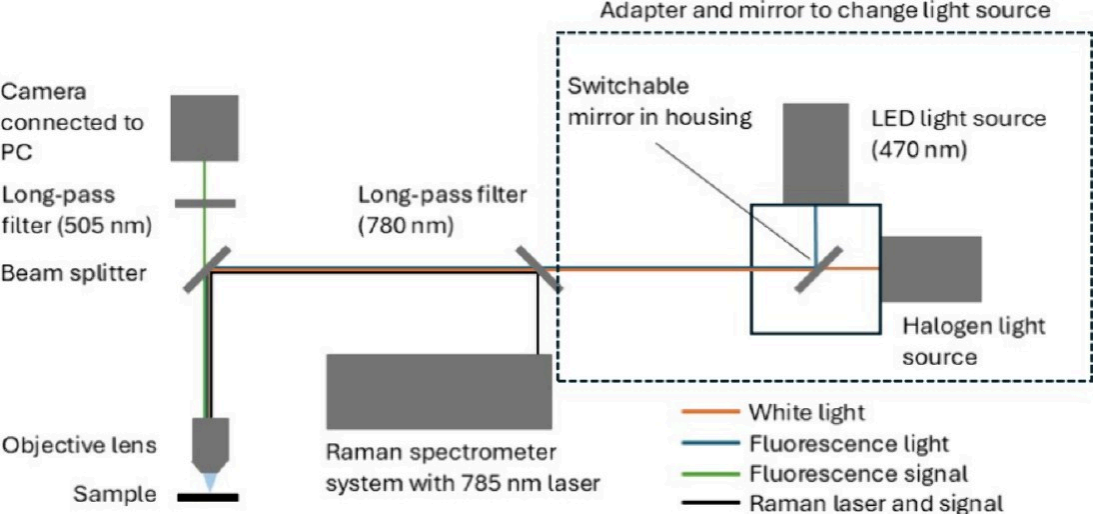
Diagram of the optical beam paths of a Raman spectrometer that utilized a microscope (left side of the image with the camera and objective lens, with a changeable beam splitter to view through the camera or eyepieces) where an additional long-pass filter is added for fluorescence monitoring. On the right of the diagram, the adapter system is highlighted in the dashed lines; a switchable mirror is used to select the light source required, and the 505 nm long-pass filter is moveable to allow full white light imaging or fluorescence only.

Raman spectroscopy measurements were conducted using the control software specific to each system. The Renishaw InVia 2000 used WiRE (version 2.1) with a 785 nm laser set at 10% power, a 5x objective, a 5-s accumulation time, and three accumulations. Only reference MPs were measured on this system due to challenges with its stage and software. As a legacy system, it is no longer fully supported, having been replaced by newer models.

The Thermo Scientific DXR2, still in production, was used to measure both reference MPs and environmental samples. The Olympus TH4–200 halogen light source is standard with this system. Using OMNIC (version 9.9), Raman spectra were collected with a 780 nm laser, a 10x objective, 20 mW at the sample, a 5-s exposure, and three accumulations.

### 2.5 Image Mapping

To test the feasibility of fluorescence-guided Raman detection and to reduce the overall time spent per filter using automated detection, the mapping function within DXR2 Raman was used in fluorescent mode which aided the operator in identifying particles stained with NR (Figures 3 and 5). The Atlμs function was used to capture brightfield images and generate particle maps. The Particle Analysis function was adjusted to ensure optimal particle selection; the automask feature was turned off, and the particle size and image intensity histograms were optimized (see Figure S4). This was then repeated in fluorescence mode and the difference in particle numbers was assessed to enable understanding of the selectiveness of fluorescence-guided detection.

**Figure 3:**
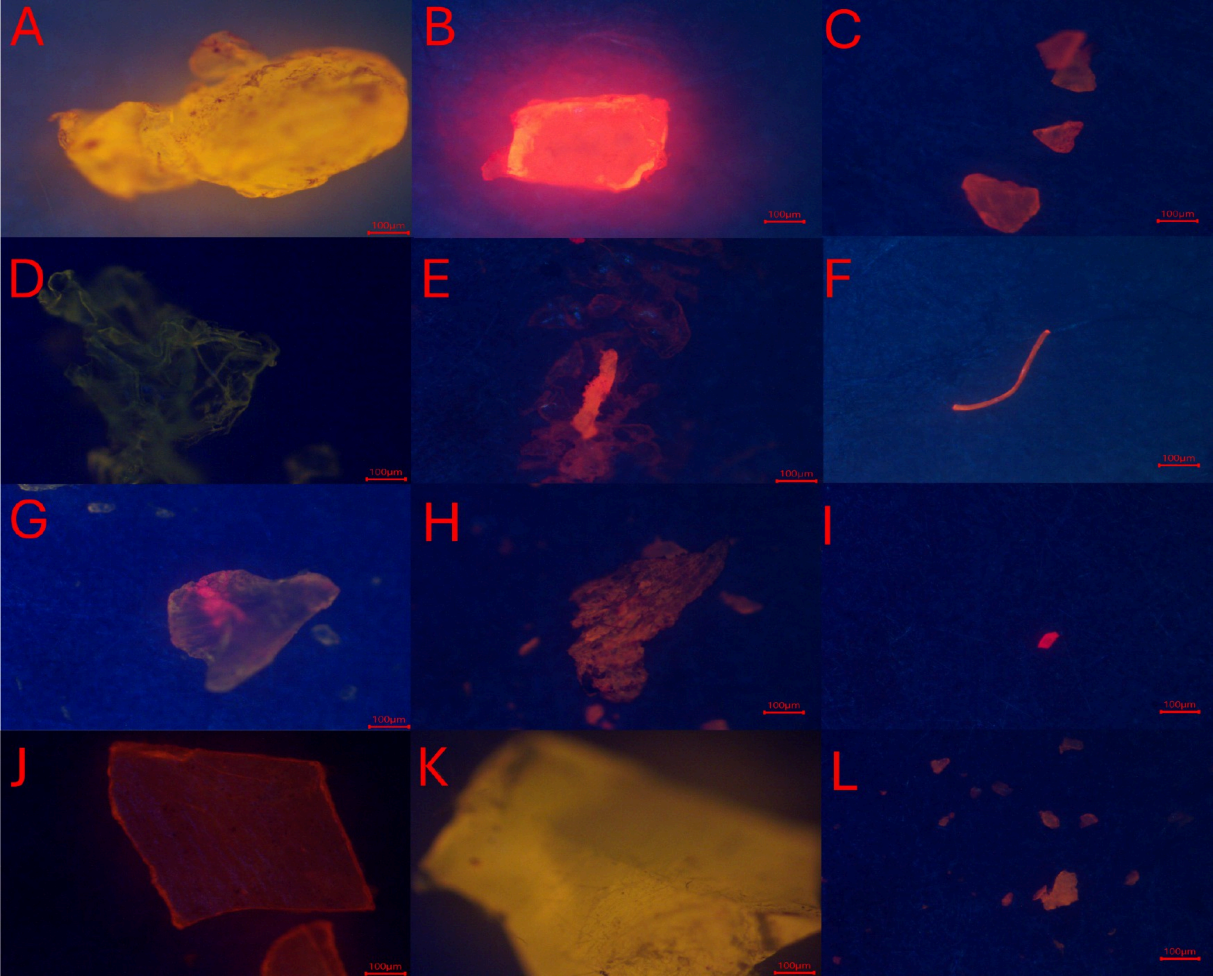
Examples of reference microplastics (prepared by grinding the reference polymers) imaged under the modified (fluorescence-guided) Raman. (A) High-density polyethylene (HDPE), (B) polyamide-6 (PA-6), (C) polyethylene (PE), (D) expanded polystyrene (EPS), (E) polyethylene terephthalate (PET), (F) polyester (PES), (G) polylactic acid (PLA), (H) polypropylene (PP: gray), (I) PP (blue), (J) polystyrene (PS), (K) polyvinyl Chloride (PVC), and (L) PP (white). Scale bars are 100 μm.

**Figure 4:**
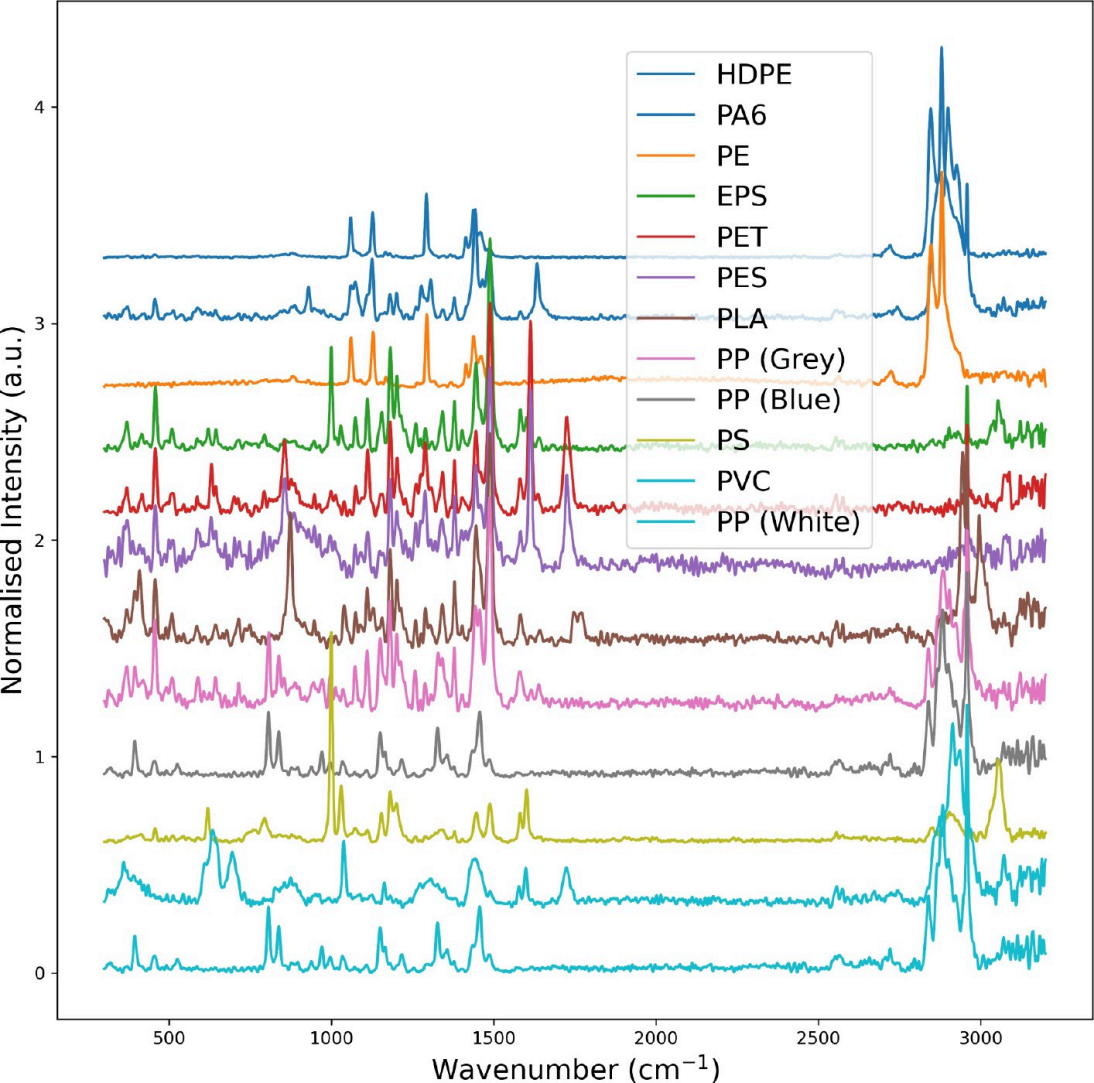
Raman spectra collected from the 12 Nile red stained reference microplastics.

**Figure 5:**
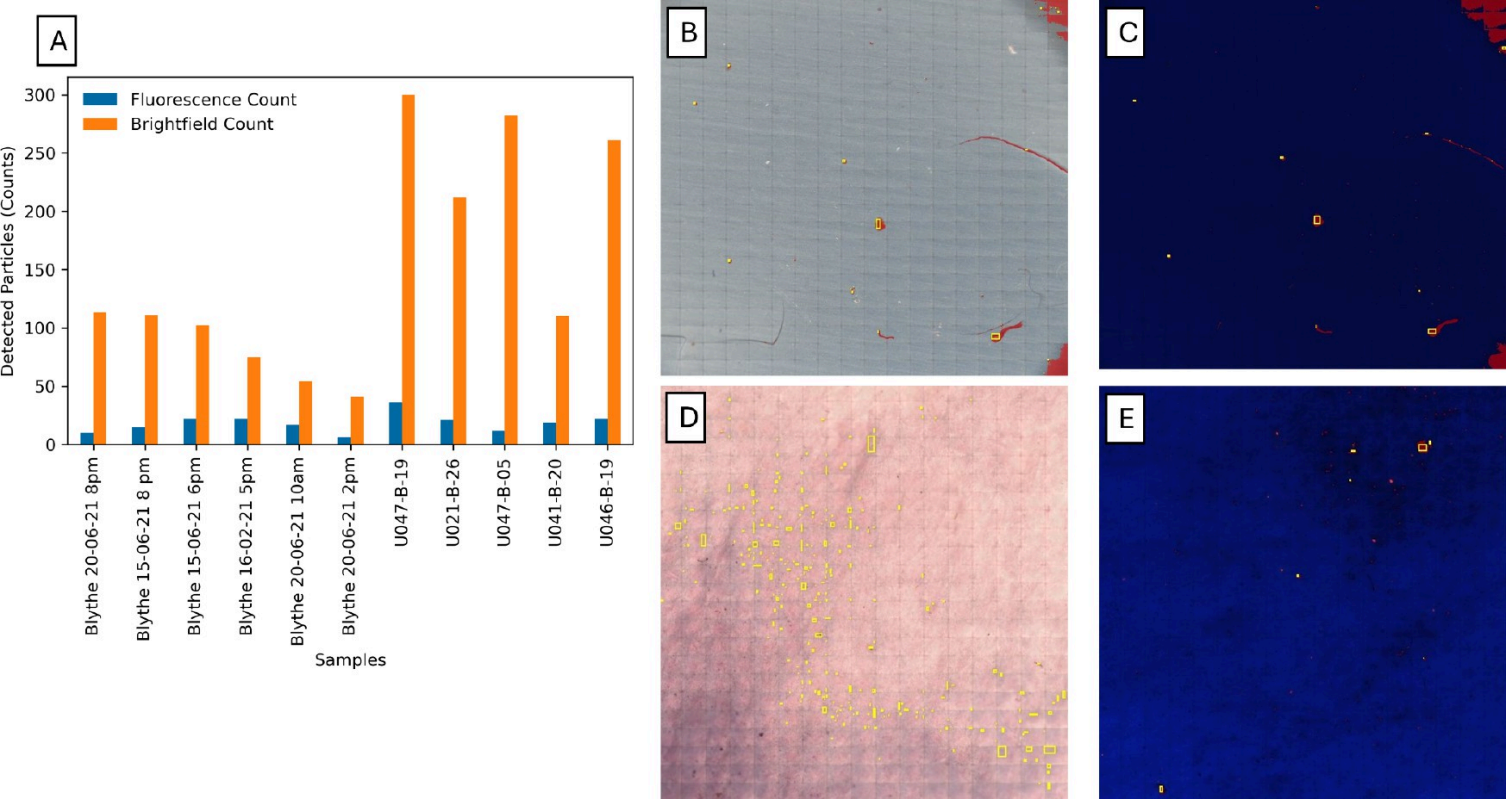
(A) Graph comparing the total number of detected particles across environmental samples: river water (Blythe) and drinking water (prefix UO). River water samples were assessed using Anodisc substrates, while drinking water samples used glass fiber GF/D substrates. (B, C) Brightfield and fluorescence detection images for Blythe 20-06-2021 2 PM, showing 41 and 6 counts, respectively. (D, E) Brightfield and fluorescence detection images for UO47-B-05, showing 282 and 12 counts, respectively. (B, C and D, E) Demonstration of the reduction of MP selected through the fluorescence signal with the particle analysis function.

### 2.6 Analysis

Raman spectra for each particle (from the reference MPs and environmental samples) were analyzed using a custom “Raman Analyzer” Python script,^24^ the baseline was removed (ZhangFit function) and peaks were matched to reference libraries, with >70% match quality index considered as positive identification. The spectral library consisted of the SLOPP, SLOPP-E^25^ and an in-house reference library, see Table S2.

## 3 Results and Discussion

### 3.1 Validation Using Microplastic Reference Materials

The 3D-printed adapter was evaluated for fit and usability on two Raman microscopes: a Renishaw InVia 2000 and a Thermo Scientific DXR2. The adapter did not hinder bright light or fluorescence illumination of the NR-stained samples under investigation and could be resolved both visually and by the software. Optical performance for fluorescence illumination and Raman signal acquisition was validated by the analysis of the 12 reference MP polymers (see Section 2.1). The polymer type and fluorescence images were previously confirmed.^20^ While slight variation in fluorescence intensity was observed between the two Raman systems (potentially due to differences in objective quality or imaging camera performance), all samples were successfully analyzed (Figure 3), and all polymer types were correctly identified (Figure 4). Common false positives tested for and identified in the samples when examined under brightfield include cellulose, cellulose acetate and cotton (see Figure S4 for Raman spectra examples). These false positives are further reduced when using Nile red fluorescence guiding, however, it may be possible that false positives for organic and inorganic matter, including sand, wood and chitin may be detected depending on the procedure used for staining.^10^

### 3.2 Validation Using Environmental Samples

In the second validation step, 11 water samples (six from the River Blythe in the UK and five from public drinking water sources in Mali) from prior studies were analyzed to evaluate the performance of the fluorescence-modified Raman system. Results were obtained using the DXR2 Raman system, which incorporates automated particle mapping. This functionality is offered by other manufacturers, typically relying on contrast-based image analysis and statistical tools for particle detection and categorization.

Under brightfield imaging, a much higher number of putative particles were detected compared to those identified using fluorescence-guided detection (Figure 5). The use of fluorescence mode significantly reduced the number of false positive detections, due to the enhanced contrast of the NR-stained MPs against the background. This reduction of nonplastic particles (sediment or other material not removed during sample processing) effectively minimized the number of particles requiring analysis for chemical identification of the polymer type, streamlining the detection process while improving accuracy by excluding nonstained (and thus nonplastic) particles. A single spectrum collection per particle with our instrument settings takes 15 s, therefore, the time saving by using fluorescence is significant as the number of particles to be analyzed increases (see Table S3). For the water samples prepared on Whatman Anodisc substrates (River Blythe samples), the mean time saving was 81% or 17 min (range 69–91%, 9–26 min), where 88 ± 7% of particles were correctly identified as MPs compared to 9 ± 3% by brightfield imaging. For samples on Whatman GF/D substrates (Mali water sources), which have a more complex woven surface, the mean time reduction was 91% or 53 min (range 83–92%, 23–68 min), where 86 ± 3% of particles were identified as MPs in fluorescence mode compared to 8 ± 2% by brightfield imaging. This means that up to 90% of the samples on the filters were nonplastic and thus that false positives were removed from the analysis pipeline through the florescence prescreening. Also note that the mean time saving increased most for GF/D filters where many more nonpolymer particles were identified. Time saving will increase further when larger areas of sample filters are analyzed. Imaging under brightfield or fluorescence showed no difference in the type of polymer found with PE, PP, PMMA, PET and PS being the main families identified. The particle counts under brightfield, and fluorescence-guided detection are detailed in Table S3.

### 3.3 Advantages of Fluorescence-Guided Raman Spectroscopy

The modified fluorescence-guided Raman spectrometer offers significant advantages in time efficiency, and analytical accuracy for the chemical identification of MPs extracted from complex environmental samples. The low-cost fluorescence adaptation of Raman microscopes makes it highly accessible for all laboratories. The adapter design can be modified for compatibility with other mounting systems, however, this was not explored within the scope of this study.

In the context of MP research, where sample numbers are often high, demanding either subsampling of the filter for spectroscopy^12,26^ or manual selection of subpopulations of the identified MPs for analysis, this hybrid fluorescence-guided Raman system proves to be a practical and cost-efficient solution. Specifically, fluorescence-enhanced particle detection increased selectivity by focusing on NR-stained particles, reducing the time that is used per filter by prescreening out false positives, while also addressing the limitations of manual handling and subsampling as these are no longer needed. The modified system also enabled whole-filter imaging in less time than the subsampling with conventional Raman, as the image mapping under fluorescent mode significantly reduced the number of particles analyzed by prescreening out the nonplastic false positive particles. This was particularly beneficial for the Whatman GF/D filters, where the reduction factor reached 90% (53 min), highlighting the potential of this method to streamline MP chemical identification workflows.

Although the integrated particle mapping and analysis platform in the Thermo Scientific DXR2 system used here facilitated efficient detection, this feature is not mandatory. Particle mapping in this platform (and others) makes use of light contrast methods to identify particle edges, meaning that in certain cases partially visible particles may be missed. The fluorescence signal coupled with the bandpass filter provides even greater contrast than brightfield particle mapping alone. Our proposed system makes use of the high contrast, where even low fluorescing particles can be identified and measured. The fluorescence-guided detection method can be adapted to Raman spectrometers without particle detection platforms by manually scanning a select area or the whole filter under fluorescent mode. This higher selectivity will reduce user bias or error in particle selection, as the fluorescent particles, even those with low fluorescence, are more easily noted compared to those in brightfield. Using this approach for manual scanning we see a typical time saving of 1–2 h or approximately 50% time saving per sample, and a reduced user error rate when carrying out quality control assessment between samples analyzed. However, this time saving will vary by system and the complexity of the samples being studied.

The ability of this method to prescreen out nonplastic particles results in improved analytical accuracy, leading to measurement times being reduced by an average of 84 ± 8% per filter (33 min), with 87 ± 9% of particles identified confirmed as MPs. A number of particles may not pass the threshold for confirmation as MPs and natural organic particles may be fluorescent which would account for not all particles being identified. Particles identified by fluorescence but not classified as polymers had a low percentage match to reference spectra when analyzed, due to their poor Raman spectra which can be a result of environmental weathering. Across both imaging approaches we noted no significant difference in type of polymer found, the main difference was attributed to the number of nonfluorescence particles which are not associated with a polymer family, often with a low signal-to-noise not discernible for spectral matching. Furthermore, the relatively low cost of the modification enables existing Raman systems to integrate fluorescence detection with Raman spectroscopy to facilitate accurate fluorescence-guided MP identification with reduced manual manipulation of the MP particles.

### 3.4 Limitations

Despite these advantages, limitations remain, particularly regarding the dependence on NR staining. The solvatochromatic nature of NR introduces variability in fluorescence based on the polymer’s microenvironment,^12,13^ which may complicate MP detection in mixed-matrix samples, and lead to some specific MP being under-represented (PET and PVC).^10,13^ However, this is not likely to lead to significant underestimation, as depending on additives present in the polymer, the same polymer will give off different fluorescent signals.^13^ While the results demonstrate the effectiveness of fluorescence-guided detection, further validation is required for different matrix types, especially those that contain complex natural organic matter that binds NR.

## 4 Conclusions

This study presents a novel fluorescence-guided Raman spectroscopy approach that directly integrates fluorescence prescreening within the Raman system through a custom adapter. Unlike previous methods that rely on external fluorescence imaging or separate prescreening steps, this modification enables seamless detection and chemical identification within a single analytical workflow. By eliminating the need for manual sample transfer and reducing nonpolymer interference before chemical validation by Raman spectroscopy, our approach significantly enhances efficiency, achieving an 84% reduction in analysis time. Additionally, the low-cost adapter design makes this method easily adaptable to existing Raman systems, improving accessibility for laboratories conducting MP research. These advancements address key challenges in MP analysis by streamlining workflows, minimizing contamination risks, and improving accuracy through fluorescence-guided particle selection. Its adaptability and cost-effectiveness make it an attractive option for laboratories seeking to enhance their MP detection capabilities and throughput. Future developments should focus on expanding the system’s compatibility, validating a wider range of polymers, and refining detection algorithms to address the remaining limitations.

## Supplementary Material



## Data Availability

Data collected for the study is available in the Supporting Information. The Adapter STL file for 3D printing is available at https://birminghamwatercentre.com/facilities/.

## Notes

The schematic diagram and measurements (in millimeters) of the custom fluorescence and white light adapter (Figure S1); the schematic diagram and measurements (in millimeters) of the custom adapter mirror slider (Figure S2); the schematic diagram and measurements (in millimeters) of the custom optics slider for the Thermo Scientific DXR (Figure S3); the image of the particle analysis function in the OMNIC software, the turned off auto mask function, and the optimized particle size and light intensity histograms (Figure S4); the Raman spectra plot of common false positive results tested for during analysis with the Raman analyzer software (Figure S5); the breakdown of item sources and costs at the time of publication (Table S1); the list of the polymer types used in the Raman database and the number of unique samples measured for each excluding the SLOPP and SLOPP-E libraries (Table S2); and the list of bright light versus fluorescence guided particle mapping detection from water samples, river Blythe, and drinking water samples from Malei (prefix UO) (Table S3) (PDF)

## Abbreviations

MP: Microplastic
NaCl: Sodium Chloride
ZnCl2: Zinc Chloride
NR: Nile Red
FTIR: Fourier Transform Infrared
HDPE: High-Density Polyethylene
PET: Polyethylene Terephthalate
PES: Polyester
PLA: Polylactic Acid
PP: Polypropylene
PS: Polystyrene
PVC: Polyvinyl Chloride
GF/D: Glass Fiber (/ grade)
