# Supplementary material for: Fluorescence-Guided Raman Spectroscopy with an Integrated Adapter for Faster and Cost-Effective Microplastic Detection
Source: Anal Chem. 2025 Nov 20;97(47):26071–8. doi: 10.1021/acs.analchem.5c04637 (PMC12676507; doi:10.1021/acs.analchem.5c04637)
Supplement: Supplementary file 1 [file ac5c04637_si_001.pdf]

Supporting Information

Fluorescence-guided Raman Spectroscopy with an Integrated Adapter for Faster and Cost-Effective Microplastic Detection

Anna Kukkola[1, †], Liam Kelleher[1, †, *], Iseult Lynch[1], Stefan Krause[1,2]

[1] School of Geography, Earth and Environmental Sciences, University of Birmingham, Edgbaston, Birmingham, B15 2TT, United Kingdom.

[2] Ecologie des Hydrosystèmes Naturels et Anthropisés (LEHNA), Université Claude Bernard Lyon 1, Lyon, 69622, Villeurbanne, France.

† Authors contributed equally.

* Corresponding author: Liam Kelleher, School of Geography, Earth and Environmental Sciences, University of Birmingham, Edgbaston, Birmingham, B15 2TT, United Kingdom. l.kelleher@bham.ac.uk

Table of Contents

Figure S1 – Schematic diagram and measurements (in millimetres) of the custom fluorescence and white light adapter. 2

Figure S2 – Schematic diagram and measurements (in millimetres) of the custom adapter mirror slider. 3

Figure S3 – Schematic diagram and measurements (in millimetres) of the custom optics slider for the Thermo Scientific DXR. 3

Figure S4 – Image of the Particle Analysis function in the OMNIC software, the Auto mask function is turned off, and the particle size and light intensity histograms are optimised. 4

Figure S5 – Raman spectra plot of common false positive results tested for during analysis with the Raman Analyzer software. 4

Table S1 – Breakdown of item sources and costs at time of publication. 5

Table S2 – List of the polymer types used in the Raman database and number of unique samples measured for each. This excludes the SLOPP and SLOPP-E libraries. 6

Table S3 – List of bright light versus fluorescence guided particle mapping detection from water samples, river Blythe and drinking water samples from Malei (prefix UO). 7

Figure S1 – Schematic diagram and measurements (in millimetres) of the custom fluorescence and white light adapter.

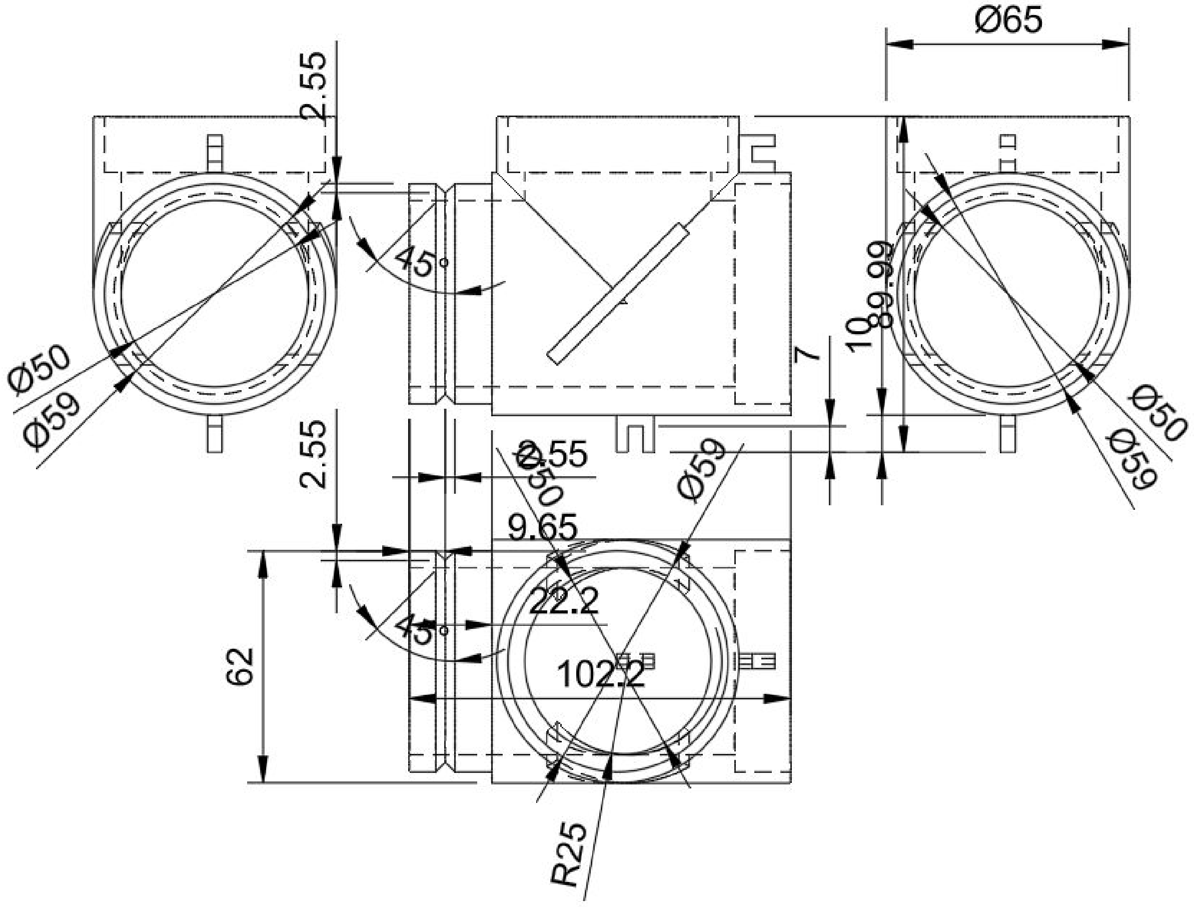

Figure S2 – Schematic diagram and measurements (in millimetres) of the custom adapter mirror slider.

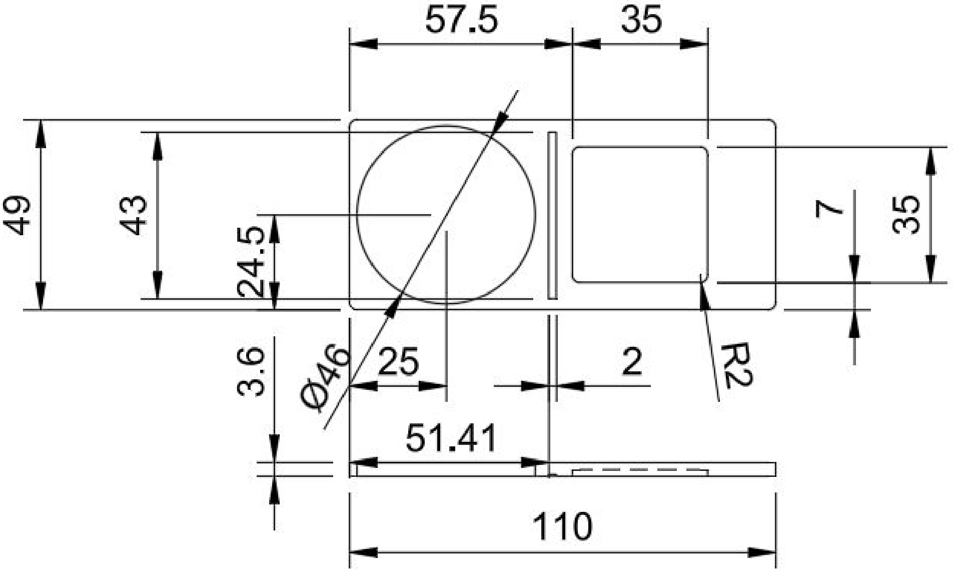

Figure S3 – Schematic diagram and measurements (in millimetres) of the custom optics slider for the Thermo Scientific DXR.

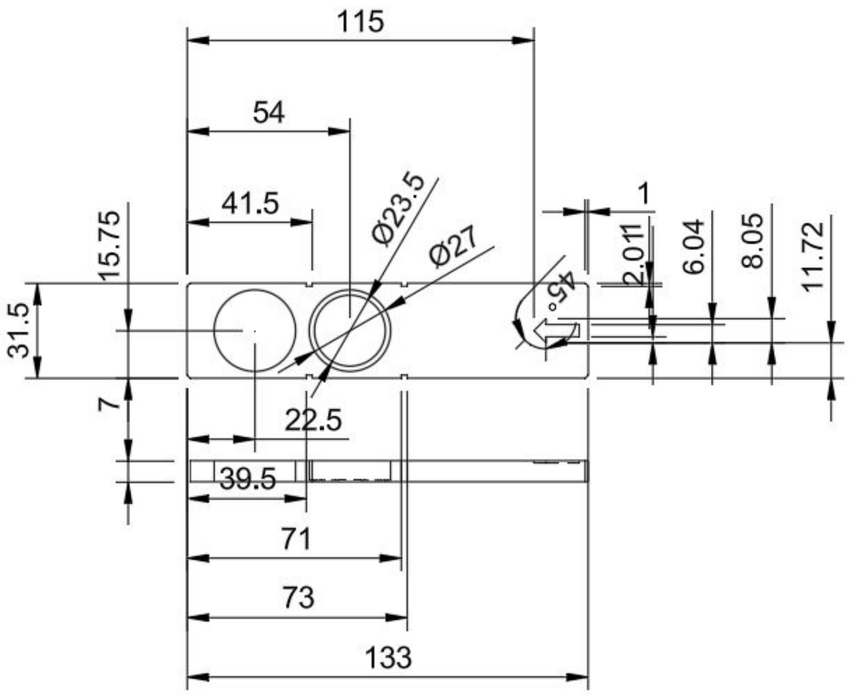

Figure S4 – Image of the Particle Analysis function in the OMNIC software, the Auto mask function is turned off, and the particle size and light intensity histograms are optimised.

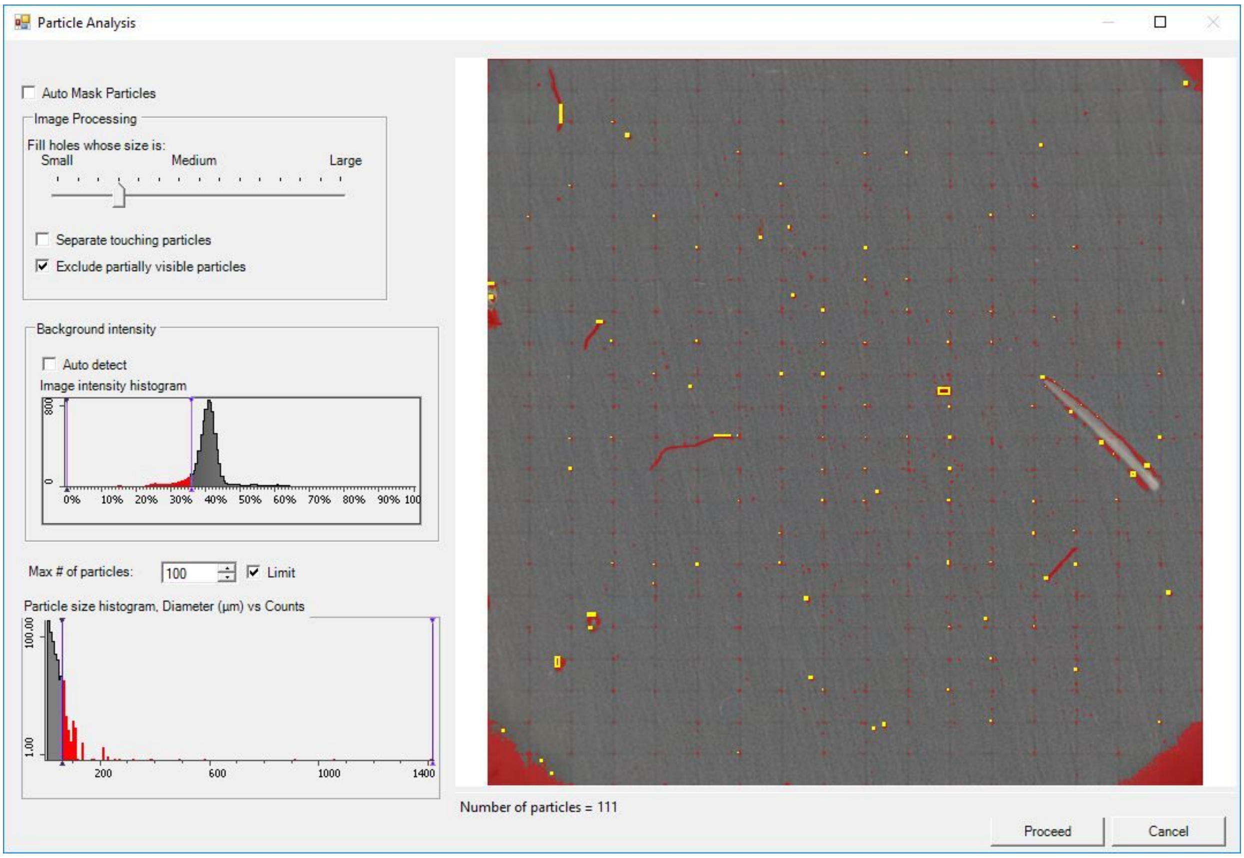

Figure S5 – Raman spectra plot of common false positive results tested for during analysis with the Raman Analyzer software.

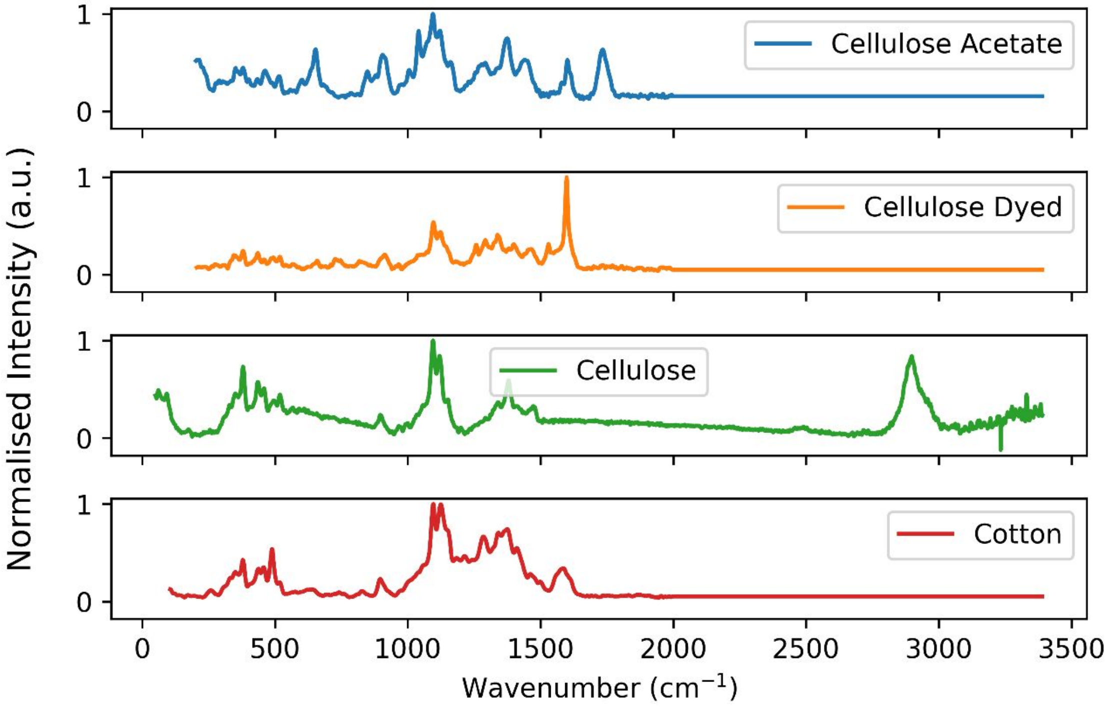

Table S1 – Breakdown of item sources and costs at time of publication.

**Item Name**	**Cost (£)**	**Link**
**Parts**		
SOLIS-470C - High-Power LED for Microscopy, 470 nm (Blue), 3.0 W (Min)	1,171.42	https://www.thorlabs.com/thorproduct.cfm?partnumber=SOLIS-470C
DC20 - High-Power Driver for Solis® LEDs, 10 A Max, 14.0 V Max	461.19	https://www.thorlabs.com/thorproduct.cfm?partnumber=DC20
DMLP505T Longpass Dichroic Mirror, 505 nm Cut-On	107.39	https://www.thorlabs.com/thorproduct.cfm?partnumber=DMLP505T
**Material**		
Ultimaker 2.85mm Black PLA 3D Printer Filament, 750g	42.60	https://uk.rs-online.com/web/p/3d-printing-materials/1348185

Table S2 – List of the polymer types used in the Raman database and number of unique samples measured for each. This excludes the SLOPP and SLOPP-E libraries.

LDPE– low density polyethylene	3
LLDPE- low-low density polyethylene	2
MDPE- medium density polyethylene	2
HDPE - high density polyethylene	4
ULDPE– ultra low density polyethylene	1
PES- polyester	2
PET- polyethylene terephthalate	6
PP- polypropylene	7
PS - polystyrene	3
PVC – polyvinyl chloride	3
PU – polyurethane	2
ABS- acrylonitrile butadiene styrene	3
PC – polycarbonate	2
PVA – polyvinyl alcohol	2
PA6 – polyamide 6/nylon	2
PA66 – Polyamide 66/Nylon	2
Cotton	4
PMMA – poly (methyl metacrylate)	3
Rubber	3
CA - Cellulose acetate	2
PTFE - polytetrafluoroethylene	2
PLA – polylactic acid	2

Table S3 – List of bright light versus fluorescence guided particle mapping detection from water samples, river Blythe and drinking water samples from Malei (prefix UO).

Sample	Fluorescence Count	Brightfield Count	Percentage Count Reduction	Time Saving (Minutes)	Fluorescence Polymer ID	Brightfield Polymer ID	Fluorescence MP Identified (%)	Brightfield MP Identified (%)
Blythe 20-06-21 8pm	10	113	91	26	9	8	90	7
Blythe 15-06-21 8 pm	15	111	86	24	14	10	93	9
Blythe 15-06-21 6pm	22	102	78	20	20	9	91	9
Blythe 16-02-21 5pm	22	75	71	13	20	6	91	8
Blythe 20-06-21 10am	17	54	69	9	15	8	88	15
Blythe 20-06-21 2pm	6	41	85	9	4	2	67	5
U047-B-19	36	300	88	66	31	26	86	9
U021-B-26	21	212	90	48	18	11	86	5
U047-B-05	12	282	96	68	11	21	92	7
U041-B-20	19	110	83	23	15	8	79	7
U046-B-19	22	261	92	60	19	31	86	12
